# Coronavirus Nsp15 endoribonuclease: linking viral RNA regulation to immune evasion and viral fitness

**DOI:** 10.1128/jvi.01700-25

**Published:** 2026-07-31

**Authors:** Xueying Liang, Xiang Chi, Xufang Deng

**Affiliations:** 1 Department of Physiological Sciences, College of Veterinary Medicine, Oklahoma State University 70729, Stillwater, Oklahoma, USA; 2 Oklahoma Center for Respiratory and Infectious Diseases, Oklahoma State University 630347, Stillwater, Oklahoma, USA; Universiteit Gent, Merelbeke, Belgium

**Keywords:** coronavirus, Nsp15, endoribonuclease, RNA processing, immune evasion, viral fitness

## Abstract

Coronavirus nonstructural protein 15 (Nsp15) is a conserved uridine-preferring endoribonuclease (EndoU). Studies using mouse hepatitis virus (MHV), SARS-CoV-2, and other coronaviruses have shown that Nsp15 associates with replication–transcription complexes (RTCs) and contributes to viral immune evasion. Structural studies of alpha- and beta-coronavirus Nsp15 proteins reveal a hexameric enzyme that engages viral RNA substrates and cleaves at unpaired uridines through an RNase A-like, largely metal-independent mechanism stimulated by divalent cations. The Nsp15 hexamer functions as a dynamic, cooperative platform capable of accommodating extended double-stranded and structured RNA substrates. Genetic studies in several coronaviruses indicate that EndoU activity is dispensable for viral RNA synthesis in cell culture, but critical for suppressing host antiviral responses. Loss of EndoU activity promotes accumulation of immunostimulatory RNA species and activation of dsRNA-sensing pathways, including MDA5-dependent interferon signaling, PKR-mediated translational arrest, and the OAS/RNase L system. Mechanistically, Nsp15 is proposed to suppress these responses by selectively processing uridine-rich and structurally accessible regions in viral RNA, including poly(U)-containing negative-strand RNAs, and elements within untranslated regions and transcription regulatory sequences. Beyond catalysis, Nsp15 may contribute to RTC organization and regulate viral RNA recombination or defective viral genome formation, although these roles remain less well-defined and may vary among coronavirus species. Together, these findings support a model in which Nsp15 functions as a regulator of viral RNA composition and immunogenicity rather than solely as a degradative nuclease. This review summarizes recent advances in Nsp15 structure, RNA processing, immune evasion, and antiviral targeting, and highlights key unresolved questions.

## INTRODUCTION

Coronaviruses (CoVs) are enveloped, positive-sense RNA viruses in the subfamily *Orthocoronavirinae*, family *Coronaviridae*, and order *Nidovirales*. They are classified into four genera: alpha-, beta-, gamma-, and delta-coronavirus, and possess some of the largest known RNA virus genomes, approximately 26–30 kb in length. Upon entry, the viral genome functions as mRNA and is translated into the replicase polyproteins pp1a and pp1ab, the latter produced through a −1 ribosomal frameshift. These polyproteins are processed by viral proteases into nonstructural proteins (Nsps), which assemble replication–transcription complexes (RTCs) on virus-induced double-membrane vesicles (DMVs) to mediate viral RNA synthesis (1, 2). Nsp15 is the 15th cleavage product of pp1ab and was first identified in human common cold coronavirus (HCoV) 229E and mouse hepatitis virus (MHV) (3, 4). Sequence analysis predicted homology between the C-terminal region of Nsp15 and a Xenopus laevis poly(U)-specific endoribonuclease, and subsequent biochemical studies showed that purified SARS-CoV Nsp15 cleaves single- and double-stranded RNA at pyrimidines, with a preference for uridines, establishing Nsp15 as a uridine-preferring endoribonuclease (EndoU) (5–7). In HCoV-229E- and MHV-infected cells, Nsp15 localizes to perinuclear puncta and colocalizes with viral RNA and RTC-associated proteins, suggesting that it acts near sites of viral RNA synthesis (3, 4, 8).

Although early studies proposed that EndoU activity contributes directly to RNA synthesis (6, 9), recombinant MHV studies showed that loss of EndoU activity does not impair viral RNA replication in interferon (IFN)-deficient cells but causes severe defects in immune-competent cells and attenuation *in vivo* (10, 11). This phenotype is associated with activation of dsRNA-induced pathways, including melanoma differentiation-associated protein 5 (MDA5)-dependent IFN induction, protein kinase R (PKR)-mediated translational arrest, and 2′−5′-oligoadenylate synthetase (OAS)/Ribonuclease L (RNase L) activation (10–12). These MHV studies, together with later work in other coronaviruses, established that a major function of Nsp15 EndoU activity is to antagonize host innate antiviral responses rather than to directly support RNA synthesis.

Recent structural and biochemical studies have defined Nsp15 as a hexameric RNase A-like enzyme that recognizes both sequence and structural features of RNA substrates, including uridine-rich regions and accessible unpaired uridines within structured RNA (13–17). Genetic and transcriptomic studies have begun to identify physiological targets, including poly(U)-containing negative-strand RNAs, transcription regulatory sequences (TRSs), and RNA species linked to aberrant recombination and immunostimulation (18–20). Together, findings from MHV, SARS-CoV-2, and other coronavirus systems support a model in which Nsp15 functions within or near the RTC not only as an endoribonuclease but also as a regulator of viral RNA composition and immunogenicity. In this review, we summarize recent advances in the structural, biochemical, and biological understanding of Nsp15, with emphasis on studies published since our previous review (21). Although Nsp15 is conserved across CoVs, the experimental evidence discussed below is based on studies of specific coronavirus species, which are indicated where appropriate.

## SEQUENCE, STRUCTURE, AND BIOCHEMICAL FEATURES OF NSP15

Since its identification as a conserved EndoU-containing protein, numerous structures of Nsp15 have defined its domain organization, oligomeric assembly, and catalytic mechanism (see reviews in references 22, 23). Here, we summarize features most relevant to RNA recognition and enzymatic activity.

### Nsp15 is a conserved coronavirus protein

**Fig 1:**
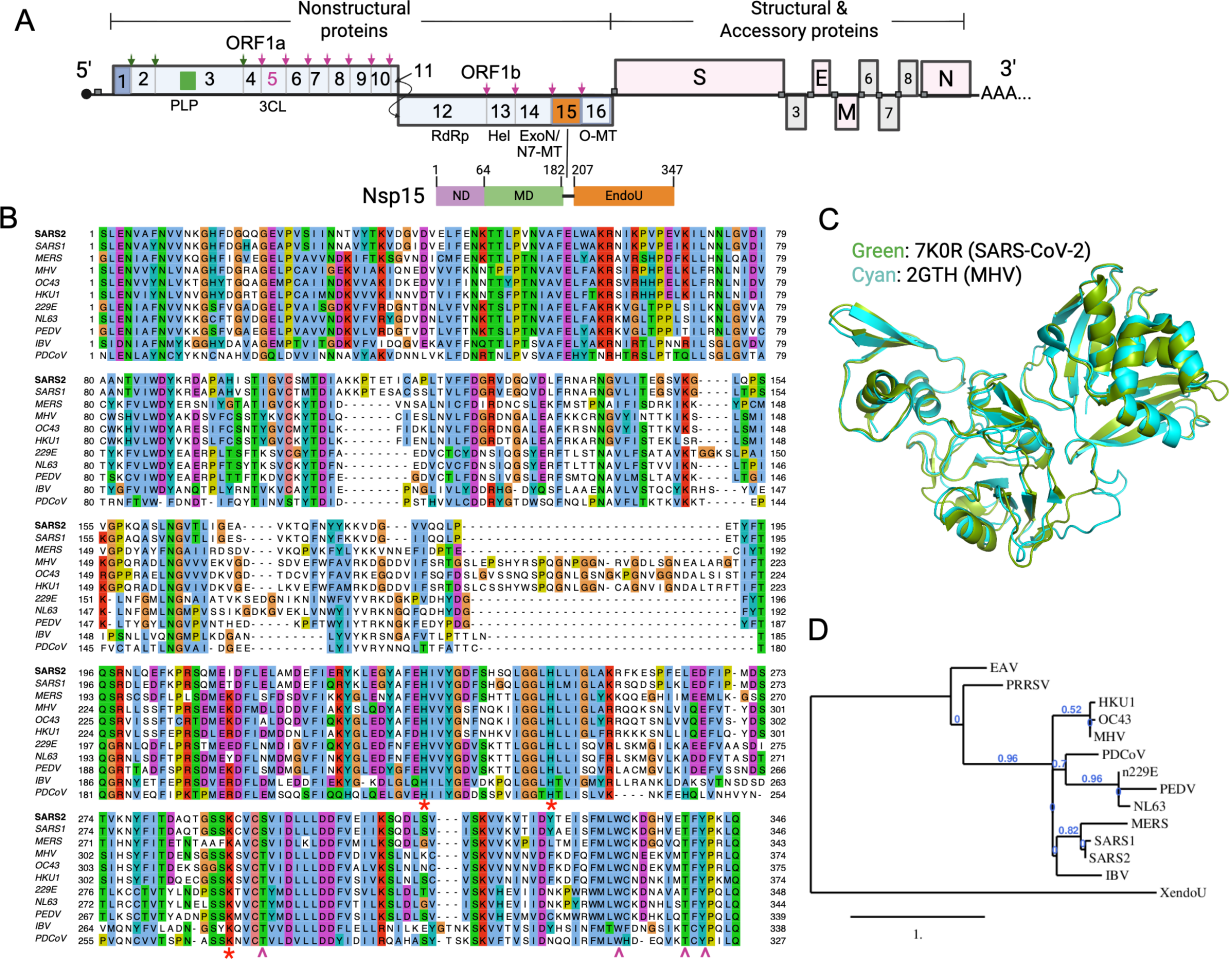
Coronavirus Nsp15 conservation. (**A**) Schematic diagram of SARS-CoV-2 genome organization. Colored arrows indicate cleavage sites recognized by viral proteases: papain-like protease PLP (green) and 3C-like protease 3CLpro (purple). E, envelope; EndoU, endoribonuclease; ExoN, exoribonuclease; Hel, helicase; M, matrix; N, nucleocapsid; N7-MT, guanosine-N7-methyltransferase; O-MT, O-methyltransferase; RdRp, RNA-dependent RNA polymerase; S, spike. (**B**) Multiple sequence alignment of Nsp15 protein sequences. Putative residues involved in catalysis (*) or substrate specificity (^). Sequence alignments were rendered and analyzed using Jalview 2.11 (26). GenBank accession numbers: SARS2 (YP_009724389), SARS1 (NP_828849), MERS (YP_009047202), MHV (NP_045299), OC43 (YP_009555238), HKU1 (YP_173236), 229E (NP_073549), NL63 (XKT23283), PEDV (AGO58929), IBV (AKV63203), and PDCoV (AKA97936). (**C**) Monomer structural comparison of SARS-CoV-2 Nsp15 (green, 7K0R) and MHV Nsp15 (cyan, 2GTH). (**D**) Phylogenetic relationship of Nidovirales EndoU homologs. *X. laevis* XendoU is included as an outgroup to root the tree. Red numerical values at the nodes indicate bootstrap support/posterior probabilities, representing the statistical confidence of the branching pattern. The scale bar indicates substitutions per site. Phylogenetic analysis was performed using the Phylogeny.fr platform (27). Sequences were aligned with MUSCLE (v3.8.31), and the phylogenetic tree was reconstructed using the PhyML program (v3.1) with the maximum likelihood method. Graphical representation of the tree was performed using TreeDyn.

Coronavirus Nsp15 is a conserved nonstructural protein of approximately 327–375 amino acids, with predicted molecular weights of 37–42 kDa depending on the viral species (Fig. 1A). Despite modest sequence conservation across coronavirus genera, Nsp15 proteins preserve the EndoU fold and catalytic residues (Fig. 1B and C). Nsp15 proteins from *embecoviruses* such as MHV, OC43, and HKU1 are generally longer, whereas delta-CoV Nsp15 proteins are shorter. The EndoU domain is also found in other nidoviruses, including *arteriviruses* such as equine arteritis virus and porcine reproductive and respiratory syndrome virus (Fig. 1D). Originally termed NendoU, this domain was considered a nidovirus genetic marker (6), although it is absent in some nidovirus families that primarily infect invertebrates (24, 25). This distribution suggests that EndoU is not universally required for nidovirus replication but may provide an advantage in vertebrate hosts, likely through effects on viral RNA processing and innate immune evasion.

### Overall structure, oligomerization, and catalysis

**Fig 2:**
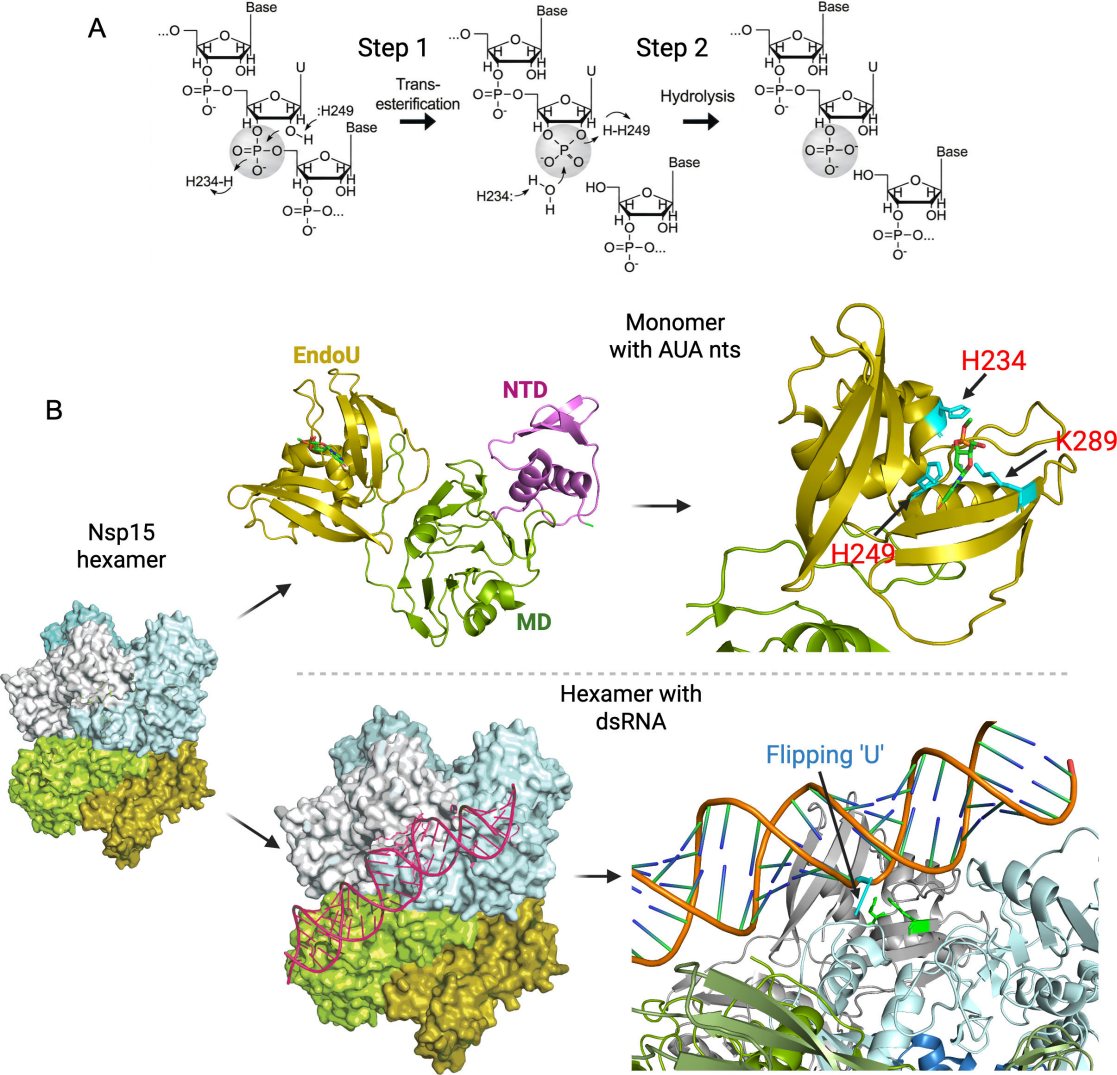
Coronavirus Nsp15 cleavage mechanism, structure, and substrate recognition. (**A**) The two-step metal ion-independent cleavage reaction is shown in schematic form. (Adapted from reference 23, previously published under a Creative Commons Attribution License.) (**B**) Structures of Nsp15. Left: Nsp15 hexamer (7TJ2). Top: structure of Nsp15 monomer bound with a tri-nucleotide AUA (7K0R), showing an NTD (purple), an MD (dark green), an EndoU domain (lemon), and a tri-nucleotide (green). The catalytic triad residues are colored in cyan with indications. Bottom: Nsp15 hexamer bound to a dsRNA molecule (7N33). One dsRNA molecule interacts with three protomers of a hexamer. A close look (bottom right) shows the targeted uridine (cyan) is dislodged and in contact with the catalytic residues (green). All structural figures were rendered using PyMOL.

Structural studies of SARS-CoV, Middle East respiratory syndrome-associated CoV (MERS-CoV), SARS-CoV-2, and other alpha- and beta-CoV Nsp15 proteins show that Nsp15 adopts a conserved three-domain architecture and assembles as a hexamer organized as a dimer of trimers (Fig. 2B) (28–32). Each protomer contains an N-terminal domain (NTD), a middle domain (MD), and a C-terminal EndoU domain. The NTD mediates oligomerization, the MD contributes to interdomain and interprotomer organization, and the C-terminal domain contains the catalytic site (28, 32). For alpha- and beta-CoV Nsp15 proteins studied to date, hexamer formation is required for efficient nuclease activity, and disruption of interprotomer contacts reduces or abolishes enzymatic function (16, 29, 31–37). Structural information for gamma- and delta-CoV Nsp15 proteins is more limited, but porcine delta-CoV (PDCoV) Nsp15 has been reported to function in a dimeric state, similar to arterivirus Nsp11, suggesting that oligomerization is broadly important even though the assembly state may differ among viral lineages (38–40).

Nsp15 cleaves RNA through an RNase A-like mechanism centered on two histidines and one lysine (H234, H249, and K289 in SARS-CoV-2). The active-site groove contains residues that contact the scissile phosphate, recognize uridine, and position neighboring nucleotides (23, 30). SARS-CoV-2 Nsp15 catalysis proceeds through transesterification to generate a 2′,3′-cyclic phosphate product, with hydrolysis to a 3′-monophosphate occurring inefficiently (Fig. 2A) (16, 23). Although early studies emphasized a requirement for Mn^2+^ ions, more recent work supports a largely metal-independent acid-base catalytic mechanism in which divalent cations enhance activity in a substrate-dependent manner, likely by stabilizing substrate engagement or catalytically favorable enzyme states (41, 42). Structural studies further indicate that the active site is dynamic: ligand or substrate binding reorganizes uridine recognition, and cleavage of duplex RNA requires base flipping to position the target uridine into the active site (13, 14, 16, 17, 30, 43). Tipiracil and related uracil analogs occupy the uridine-binding pocket, highlighting the role of residues such as S293, Y343, and W332 in base recognition and adjacent nucleotide positioning (16, 43). These structures also show that the catalytic pocket is embedded within a flexible recognition environment rather than acting as an isolated active site, a feature that may explain why RNA length, folding, and nucleotide context strongly influence cleavage efficiency.

### RNA recognition, substrate specificity, and allosteric regulation

*In vitro* studies show that coronavirus Nsp15 is a uridylate-specific endoribonuclease whose activity is shaped by sequence, local RNA structure, and oligomeric organization. Defined RNA substrates are preferentially cleaved at uridines, particularly when followed by purines, and structural studies identify a uridine-binding pocket that accommodates the base while surrounding residues help position adjacent nucleotides (16, 33, 37, 43–46). Cleavage efficiency varies with RNA length, sequence composition, and structural accessibility, indicating that uridine identity alone is insufficient to define substrate susceptibility.

RNA structure is also important for substrate recognition. SARS-CoV-2 Nsp15 cleaves both single-stranded and double-stranded (ds) RNA substrates *in vitro*, but duplex cleavage requires extrusion of the target uridine from the helix, a process favored by local flexibility (13, 17, 44, 45, 47). Cryo-EM structures of SARS-CoV-2 Nsp15 bound to dsRNA show that the substrate contacts multiple protomers across the hexamer and spans all three domains, demonstrating that higher-order assembly creates an extended RNA-binding platform (17). Ligand binding reduces conformational flexibility of the EndoU domains and promotes a more ordered state, consistent with allosteric communication across protomers (16, 30, 48). Thus, Nsp15 RNA binding, conformational ordering, and catalysis are coupled processes. The multi-protomer binding mode provides a structural basis for cooperative behavior because substrate engagement at one face of the hexamer can influence the positioning of neighboring catalytic domains. Molecular dynamics and ligand-bound structures further suggest that RNA or small-molecule binding stabilizes subsets of conformations within the hexamer, potentially regulating access to catalytically competent states (15, 16, 30, 48). These features support a model in which Nsp15 functions as an RNA-processing module that coordinates substrate access and cleavage outcomes during infection.

## BIOLOGICAL FUNCTIONS OF NSP15 DURING CORONAVIRUS INFECTION

### Association with the RTC

**Fig 3:**
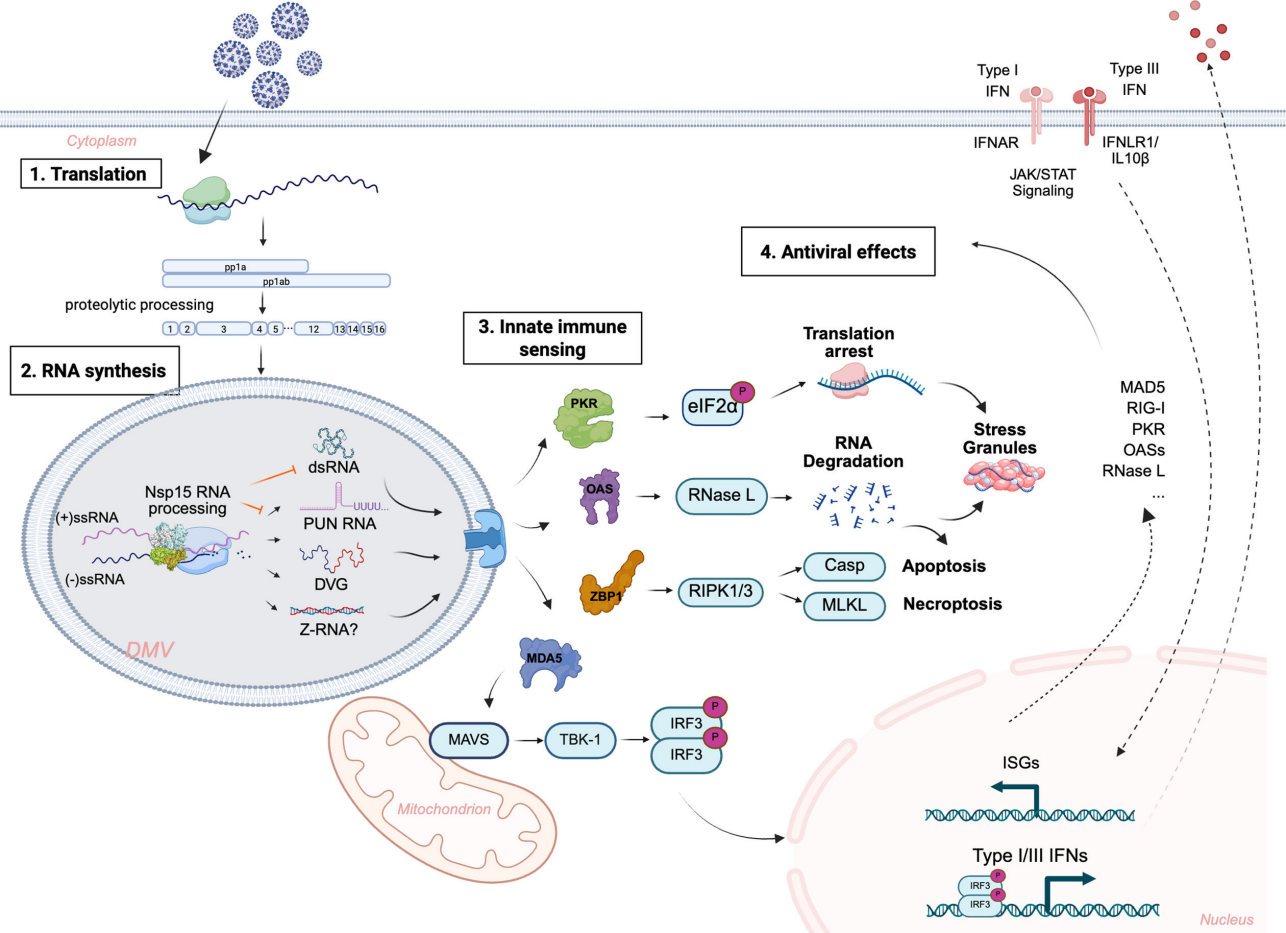
Working model for coronavirus Nsp15-mediated immune antagonism. Upon entry, the viral genomic RNA is translated to produce replicase polyproteins pp1a and pp1ab, which are proteolytically processed into individual nonstructural proteins (Nsps). Within DMVs, the replication–transcription complex (RTC) synthesizes (−)gRNA and (−)sgRNAs, which serve as templates for progeny (+)gRNA and (+)sgRNA synthesis. During this process, Nsp15 exerts critical RNA-processing functions, including limiting dsRNA accumulation, shortening the poly(U) tracts of (−)RNAs, suppressing non-canonical recombination that leads to defective viral genomes (DVGs), and potentially preventing the formation of Z-form dsRNA. In the absence of Nsp15 activity, these immune-stimulatory RNA species are released from the DMVs and recognized by various host dsRNA sensors (e.g., MDA5, PKR, OAS, and ZBP1). This sensing triggers multiple antiviral signaling cascades and cell death pathways, including type I/III interferon production, PKR-mediated translation arrest, OAS-RNase L-mediated RNA decay, and ZBP1-dependent necroptosis. Figure created with BioRender.com.

Coronavirus RTCs synthesize negative-sense genomic and subgenomic RNAs that serve as templates for progeny positive-sense RNAs (49, 50). These reactions occur within virus-induced DMVs generated by membrane-associated Nsps, which help organize RNA synthesis and may shield replication intermediates from host sensors (51–53). Multiple lines of evidence indicate that Nsp15 is closely associated with this replication environment (Fig. 3). In MHV- and HCoV-229E-infected cells, Nsp15 colocalizes with RTC markers such as Nsp8 and Nsp12 (3, 4, 8), and similar localization has been reported for infectious bronchitis virus (IBV) (54). This infection-associated distribution differs from the diffuse localization of ectopically expressed Nsp15, indicating that viral replication context strongly influences Nsp15 localization (54, 55).

Biochemical studies further support the RTC association. SARS-CoV and MERS-CoV Nsp15 interact with Nsp8 or the Nsp7/Nsp8 complex in yeast two-hybrid, pull-down, and microscale thermophoresis assays (29, 56). Nsp8 and Nsp12 can also be co-immunoprecipitated with Nsp15 in an RNA-independent manner, supporting incorporation into the RTC protein network rather than association with RNA alone (8). Finally, Nsp15 is associated with viral RNA in infected cells: MHV Nsp15 colocalizes with newly synthesized viral RNA (4), and IBV Nsp15 immunoprecipitation followed by RNA sequencing identified genomic and subgenomic viral RNAs (54). Recent modeling places the Nsp15 hexamer adjacent to the polymerase module within a larger replication superstructure, consistent with biochemical and imaging data and with structural studies showing that one Nsp15 hexamer can engage one dsRNA molecule (17, 57). Collectively, these findings suggest that Nsp15 is positioned to process viral RNA near or within the RTC. This positioning would allow Nsp15 to act on nascent or newly released replication intermediates before they accumulate or become accessible to host sensors. However, whether Nsp15 is stably incorporated into the RTC or transiently recruited to specific RNA species remains unresolved.

### Role in antagonizing host innate antiviral immunity

Given its association with RTCs and viral RNA, Nsp15 EndoU activity was initially proposed to directly promote viral RNA synthesis (6). However, recombinant MHV EndoU mutant studies have shown that EndoU activity is largely dispensable for RNA replication but important for antagonizing host innate immune responses (9–11). In 2017, Kindler et al. and Deng et al. independently showed that MHV EndoU mutants replicate poorly in mouse bone marrow-derived macrophages (BMDMs), a defect restored in type I IFN receptor knockout (*ifnar^−/−^*) BMDMs (10, 11). These mutants trigger early MDA5-dependent IFN induction, PKR-mediated translational arrest, and OAS/RNase L activation. *In vivo*, they are highly attenuated in wild-type (WT) C57BL/6 mice but exhibit pathogenicity similar to wild-type MHV in *ifnar^−/−^* mice, indicating that EndoU activity is a major fitness determinant of MHV in immune-competent hosts (10).

Subsequent studies in representative viruses from all four coronavirus genera support a broadly conserved but context-dependent role for EndoU in innate immune evasion. In IFN-deficient or innate immune-compromised cell lines, EndoU mutants often replicate similarly to WT viruses, whereas in immune-competent epithelial cells, primary cells, organoids, and animal models, they frequently show restricted replication, increased dsRNA accumulation, enhanced type I/III IFN and IFN-stimulated gene (ISG) induction, translational inhibition, and sometimes increased cell death. HCoV-229E and porcine epidemic diarrhea virus (PEDV) EndoU mutants follow this pattern, whereas the EndoU mutants of transmissible gastroenteritis virus (TGEV) and feline infectious peritonitis virus (FIPV) show minimal phenotypes in several immune-competent systems, suggesting that additional viral factors or virus-host context may compensate for loss of EndoU activity (11, 58–60). MERS-CoV provides another example of redundancy: inactivation of EndoU alone has limited impact on IFN induction or replication in A549-DPP4 cells, whereas combined disruption of EndoU with NS4a or NS4b strongly activates dsRNA-induced pathways and impairs replication (61). This reflects the roles of NS4a as a dsRNA-binding protein and NS4b as a phosphodiesterase that antagonizes OAS/RNase L (61–63). In gamma- and delta-CoVs, including IBV and PDCoV, EndoU mutants show reduced replication, enhanced IFN responses, and attenuation in animal models, with IBV EndoU activity linked to suppression of PKR activation and stress responses (54, 64–66).

SARS-CoV-2 EndoU mutants also reveal immune-dependent phenotypes. They replicate efficiently in IFN-deficient Vero cells but induce type I/III IFN responses in Calu-3 and A549-derived cell cultures, with the extent of attenuation varying across studies and conditions (20, 67–70). In primary airway cultures and organoids, EndoU mutants consistently increase IFN and ISG induction and reduce viral replication, and inhibition of IFN signaling with JAK1/2 inhibitors or STAT1 disruption restores replication, albeit with delayed kinetics (67, 68, 70). *In vivo*, SARS-CoV-2 EndoU mutants are attenuated in K18-hACE2 mice, with reduced titers, decreased pathology, and improved survival (67, 68, 71). Lung qPCR and bulk RNA-seq show increased expression of antiviral, immune effector, and inflammatory genes following mutant infection compared with WT virus (67, 71). In Syrian hamsters, EndoU mutants also induce early antiviral/inflammatory responses and show reduced viral burden at later time points, but pathology can be comparable to or greater than WT infection (20, 68, 69). Thus, enhanced immune activation appears to be a shared consequence of Nsp15 EndoU inactivation, whereas pathological outcome differs by host model, likely reflecting differences in replication kinetics, tissue tropism, innate sensing, and inflammatory responses. This interpretation is consistent with direct comparisons showing persistent alveolar infection, extrapulmonary spread, and fatal disease in K18-hACE2 mice but transient bronchial infection and reversible pulmonary disease in hamsters (72, 73).

Naturally occurring SARS-CoV-2 variation provides a complementary perspective. Population-level genomic analyses suggest that Nsp15 substitutions may influence viral fitness. The Omicron-associated T112I substitution was predicted from millions of genomes to increase fitness and was shown biochemically to enhance EndoU activity, whereas the naturally occurring catalytic-site substitution H234Y eliminates EndoU activity and has remained rare in circulating viruses (17, 36, 74). Although direct testing in recombinant viruses and relevant infection models is needed, these observations suggest that Nsp15 activity may be subject to selective pressure during SARS-CoV-2 evolution. Together, available evidence indicates that Nsp15 EndoU activity is especially important for suppressing host antiviral responses, although the extent of its contribution to viral fitness varies with infected cell type, innate immune competence, target tissue, and the presence of additional viral immune antagonists. This context dependence is important for interpreting both attenuation studies and inhibitor studies, because the consequences of EndoU disruption are likely to be underestimated in systems that lack intact IFN or RNA-sensing pathways.

### Mechanism of Nsp15/EndoU-mediated immune evasion

Host cells detect coronavirus infection through pattern recognition receptors that recognize viral RNA species, particularly dsRNA intermediates generated during replication (75–79). Although coronaviruses produce abundant dsRNA, they often induce weak or delayed IFN responses, reflecting sequestration of replication intermediates within DMVs, RNA capping and modification, and direct antagonism by viral proteins (80, 81). Within this framework, Nsp15 acts upstream of host sensing by limiting the accumulation or exposure of RNA species that activate antiviral pathways (Fig. 3).

EndoU mutant infections activate several dsRNA-induced pathways. The MDA5-IFN axis is prominent: MHV EndoU mutants induce rapid type I IFN and ISG expression, which is significantly reduced in MDA5^−/−^ or MAVS^−/−^ BMDMs (10, 11). In SARS-CoV-2, MDA5 also drives IFN induction, although its contribution to direct viral restriction varies across systems (78, 82). The PKR-eIF2α pathway is also activated after loss of EndoU activity. MHV and HCoV-229E EndoU mutants induce PKR activation and eIF2α phosphorylation, and PKR inhibition reduces early apoptosis in infected macrophages (10, 11). Similar activation has been reported for IBV, whereas in MERS-CoV, robust PKR activation is most evident when EndoU inactivation is combined with disruption of NS4a (61, 65). SARS-CoV-2 EndoU deficiency increases PKR phosphorylation, although downstream eIF2α responses are model-dependent (67, 70).

EndoU loss also enhances OAS/RNase L activation. During MHV infection, loss of EndoU increases RNase L-dependent ribosomal RNA (rRNA) degradation (10, 11). In MERS-CoV, activation of this pathway requires combined disruption of EndoU and NS4b, consistent with the ability of NS4b to counteract OAS/RNase L (61). In SARS-CoV-2, classical rRNA degradation is less consistent, but markers such as PABPC1 (Poly(A)-Binding Protein Cytoplasmic 1) nuclear translocation support increased RNase L activity during EndoU mutant infection (67, 70, 83). Additional pathways may also contribute. MHV EndoU mutants enhance ZBP1 (Z-DNA-binding protein 1) activation and inflammatory cell death in BMDMs, suggesting accumulation of RNA species capable of engaging Z-RNA sensing pathways, although the identity and origin of these RNAs remain unclear (84–87). SARS-CoV-2 EndoU mutants also show partial rescue by STING (Stimulator of Interferon Gene) knockout or inhibition in IFN-competent cells, suggesting broader effects on infection-induced nucleic acid sensing (69). Overall, EndoU activity does not target a single immune pathway; rather, it reduces the pool or accessibility of immunostimulatory RNA species that feed into multiple downstream antiviral responses. This broad effect also explains why individual pathway knockouts often provide only partial rescue: MDA5, PKR, OAS/RNase L, ZBP1, and potentially DNA-sensing pathways can operate in parallel or sequentially, depending on the virus, cell type, and kinetics of infection.

### Physiological targets of Nsp15 EndoU

The activation of multiple RNA-sensing pathways during EndoU mutant infection indicates that Nsp15 targets viral RNA species that would otherwise function as pathogen-associated molecular patterns (PAMPs). EndoU mutant infections are associated with increased dsRNA or dsRNA-like species, as detected with dsRNA-specific antibodies in MHV, MERS-CoV, SARS-CoV-2, and IBV systems (11, 54, 61, 68, 70). Because dsRNA is also detected during WT infection and is likely sequestered within DMVs, excess dsRNA puncta in EndoU mutant MHV that fail to colocalize with DMV markers suggest accumulation or mislocalization of RNA species accessible to host sensors (18, 51, 52, 88). The identity of these species remains complex. RNA immunoprecipitated with dsRNA antibodies during MHV infection is enriched for negative-sense RNA rather than canonical duplex RNA, raising the possibility that antibodies detect structured single-stranded RNAs or dsRNA-like conformations (18, 89).

A major advance was the identification of poly(U)-containing negative-strand RNAs (PUN RNAs) as physiological Nsp15 substrates. These RNAs arise when poly(A) tails of positive-sense genomic and subgenomic RNAs are copied into uridine-rich tracts at the 5′ ends of negative-strand RNAs (90, 91). In EndoU mutant infections, these poly(U) tracts remain extended and accumulate, correlating with enhanced MDA5-dependent IFN activation (18). Biochemical studies confirm that Nsp15 directly cleaves poly(U)-containing substrates *in vitro* (14, 16), supporting the idea that PUN RNA is a direct substrate. By shortening uridine-rich regions, Nsp15 likely reduces their immunostimulatory potential and limits recognition by host RNA sensors. A useful analogy is the hepatitis C virus, in which a long poly(U/UC) tract contributes to RIG-I recognition (92, 93). Although MDA5 rather than RIG-I appears to dominate coronavirus sensing, these examples suggest that long uridine-rich regions can serve as non-self RNA signatures. Because coronavirus replication repeatedly generates poly(A)-tailed positive-strand RNAs and their negative-strand complements, PUN RNAs may represent a recurring source of immunostimulatory RNA that EndoU activity helps control.

Transcriptome-wide mapping has further shown that Nsp15 targets multiple viral RNA regions. Using cyclic phosphate cDNA sequencing, Ancar et al. mapped MHV EndoU cleavage products and found preferential cleavage on the 3′ side of pyrimidines, especially at UA and CA junctions, with sites distributed across the genome, including untranslated regions (UTRs) and coding regions (19). Cleavage was enriched near tandem CA sequences adjacent to the MHV poly(A) tail, supporting a model in which EndoU limits dsRNA accumulation by regulating negative-strand synthesis, although this interpretation remains debated because total negative-sense RNA read counts were comparable between WT and EndoU mutant infections in another study (18). Nsp15 also targets TRS regions, which are A/U-rich structured elements involved in discontinuous transcription (19). In SARS-CoV-2, loss of EndoU activity is linked to reduced canonical TRS-mediated recombination while increasing non-canonical recombination and microdeletions, events that may generate defective viral genomes with immunostimulatory potential (20, 94). Together, these findings indicate that Nsp15 does not simply degrade canonical long dsRNA; instead, it shapes the viral RNA landscape by processing uridine-rich and structurally accessible RNA species, including PUN RNAs, UTR/TRS elements, and RNAs prone to aberrant recombination.

### EndoU-independent roles of Nsp15 in viral RNA synthesis

Although reduced RNA accumulation in EndoU mutants is often attributable to activation of host antiviral pathways, several observations suggest that Nsp15 may also contribute to RNA synthesis through mechanisms independent of catalytic activity. First, Nsp15 interacts with RTC components such as Nsp8 and Nsp12 in an RNA-independent manner, and modeling studies place the Nsp15 hexamer within higher-order RTC assemblies, raising the possibility of a scaffold or organizational role (8, 29, 57). Consistent with this idea, deletion of Nsp15 in a SARS-CoV-2 replicon system markedly reduces viral RNA levels (95).

Second, mutagenesis studies indicate that regions outside the catalytic center can be required for replication. In MHV, catalytically inactive EndoU mutants are viable, whereas the D324A mutation prevents virus recovery and produces insoluble protein when expressed in *E. coli*, suggesting a role in folding or stability (9). In TGEV, catalytically inactive mutants are viable, but an N-terminal deletion that disrupts hexamer formation is lethal (60). SARS-CoV-2 studies also reveal complexity: dual catalytic histidine mutants reduce replicon RNA levels, and some mutations at H249 or neighboring residues prevent virus recovery, whereas H249R is recoverable despite lacking EndoU activity (68, 95, 96). Because analogous catalytic mutants have been recovered in MHV, PEDV, and TGEV (11, 60, 97), these phenotypes may reflect virus-specific structural constraints of Nsp15 rather than a universally conserved catalytic requirement for RNA synthesis.

Third, pharmacological studies support EndoU-independent functions. EPB-113 inhibits SARS-CoV-2 replication in IFN-responsive and nonresponsive cells but does not inhibit EndoU activity in biochemical assays; instead, it enhances cleavage by purified Nsp15 proteins of HCoV-229E and SARS-CoV-2 and destabilizes the hexamer (96). Resistance mutations map to the catalytic core or adjacent RNA-binding groove, and recombinant viruses carrying these substitutions display altered immune phenotypes and fitness costs (96). These findings support a model in which perturbation of Nsp15 structure, stability, or oligomeric organization can impair viral replication and immune evasion without direct catalytic inhibition. Overall, Nsp15 likely contributes to viral RNA synthesis through non-catalytic functions involving protein-protein or protein-RNA interactions, structural integrity, and higher-order RTC organization. Defining these roles will require the use of separation-of-function mutants and high-resolution analyzes of the RTC architecture.

## NSP15 AS A THERAPEUTIC TARGET

### Rationale for Nsp15 as an antiviral target

**Fig 4:**
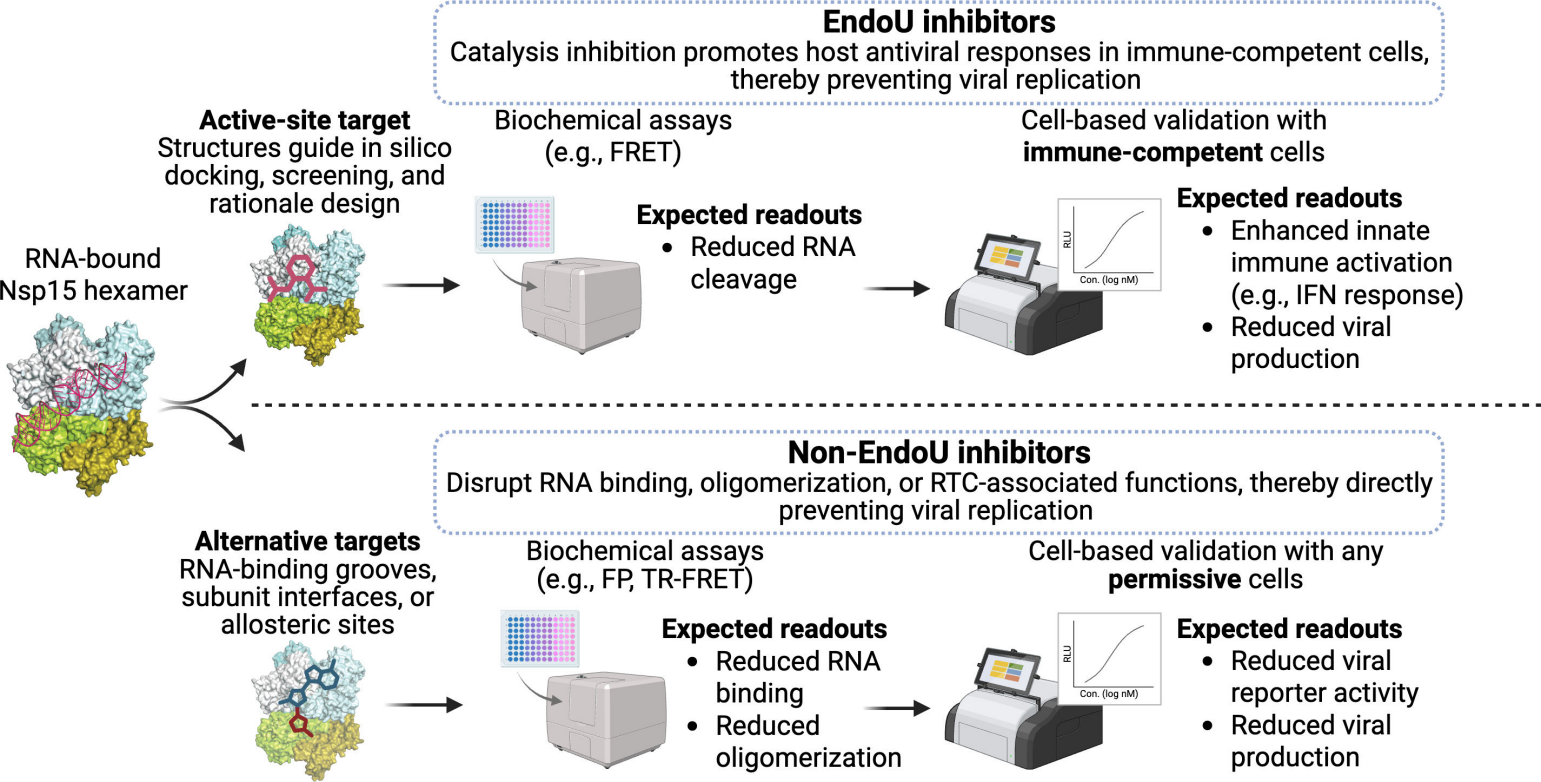
Screening and validation pipelines for EndoU and non-EndoU Nsp15 inhibitors. Schematic representation of the parallel strategies used to identify and validate small-molecule inhibitors targeting the SARS-CoV-2 Nsp15 endoribonuclease. (Top) EndoU catalytic site inhibition. Structural data guide *in silico* docking and rational design to target the active site of the Nsp15 monomer/hexamer. High-throughput biochemical assays (e.g., FRET-based molecular beacons) monitor the direct reduction of RNA cleavage activity. Cell-based validation is conducted in immune-competent cells to evaluate whether compound treatment restores host dsRNA sensing, enhances innate immune activation (e.g., interferon responses), and reduces downstream viral production. (Bottom) Non-EndoU allosteric and interface inhibition. Screen targets are directed away from the catalytic pocket toward alternative sites, including RNA-binding grooves, monomer-monomer subunit interfaces, or distinct allosteric pockets on the assembled hexamer. Screening utilizes biophysical and biochemical assays (e.g., FP, TR-FRET, and MST) to detect the disruption of absolute RNA binding or higher-order oligomerization. Cellular validation is carried out in any standard permissive cell line to confirm the reduction of viral reporter activity or a reduction in viral growth. Con., concentration; EndoU, endoribonuclease; FP, fluorescence polarization; FRET, Förster resonance energy transfer; IFN, interferon; MST, microscale thermophoresis; RLU, relative luminescence units; RTC, replication–transcription complex; TR-FRET, time-resolved Förster resonance energy transfer. Figure created with BioRender.com.

Nsp15 has attracted attention as a potential antiviral target because of its conserved uridine-specific EndoU activity and its role in limiting the accumulation of immunostimulatory viral RNA. Disruption of EndoU activity enhances innate immune responses and reduces viral fitness in immune-competent systems, providing a rationale for therapeutic targeting. Accordingly, multiple studies have sought Nsp15 inhibitors through high-throughput screening, structure-guided design, drug repurposing, and natural-product libraries. These efforts have yielded diverse compounds capable of inhibiting EndoU activity in biochemical assays, including nucleoside analogs, small molecules, covalent fragments, and natural products. Previous reviews, including Van Loy et al. (22), have summarized these discovery efforts in detail. Despite this progress, biochemical inhibition of EndoU activity has not consistently translated into antiviral efficacy. Many compounds that inhibit Nsp15 *in vitro* show weak or inconsistent antiviral activity in cell culture, particularly in IFN-deficient systems such as Vero cells. Conversely, some Nsp15-targeting compounds impair replication without inhibiting EndoU catalysis, suggesting that Nsp15 can also be targeted through effects on stability, oligomerization, RNA binding, or other functional states. These observations indicate that Nsp15 function during infection cannot be explained solely by catalytic cleavage activity. Here, we summarize key lessons from inhibitor studies and consider how they inform antiviral strategies (Fig. 4).

### EndoU inhibitors

Early studies already showed that biochemical inhibition of Nsp15 does not necessarily translate into robust antiviral activity. Because Nsp15 and RNase A share a related catalytic mechanism, Ortiz-Alcantara et al. tested RNase A inhibitors and found that benzopurpurin B inhibited SARS-CoV Nsp15 protein but showed only modest antiviral activity in Vero cells (98). Similarly, Tipiracil binds the uridine-recognition pocket and inhibits SARS-CoV-2 Nsp15 cleavage *in vitro* but has limited antiviral activity in infected cells (43), and the selective *in vitro* inhibitor NSC95397 failed to suppress SARS-CoV-2 replication in Vero E6 cells (99). These examples suggest that the antiviral activity of inhibitors with modest enzymatic inhibition may not be revealed in systems that do not capture the immune-modulatory role of Nsp15.

Several reported Nsp15 inhibitors show antiviral activity, but the contribution of Nsp15 inhibition is often difficult to define. EGCG, baicalin, and quercetin have been identified as potential Nsp15 inhibitors, but EGCG likely acts on multiple targets, including 3CLpro and viral entry (100–102). Drug-repurposing studies also identified atovaquone and pibrentasvir as direct Nsp15 inhibitors with activity against SARS-CoV-2 and HCoV-OC43, but pibrentasvir also inhibits the Nsp14/Nsp10 exonuclease, and only atovaquone induced dsRNA changes consistent with Nsp15 inhibition (103, 104). More recent screening and structure-guided studies have identified compounds with improved biochemical or cellular activity, including Exebryl-1, IPA-3, hexachlorophene, CID5675221, YM-155, TAS-103, isatin-based inhibitors, thiazolidinedione/rhodanine derivatives, and acrylamide-based covalent fragments (105–109). Collectively, these studies show that Nsp15 is chemically tractable, but further optimization of potency, selectivity, cellular target engagement, and validation in IFN-competent systems will be required for EndoU inhibitors to achieve meaningful antiviral efficacy. A practical challenge is that enzymatic IC_50_ values alone may be poor predictors of cellular antiviral activity. Assays that measure dsRNA accumulation, IFN/ISG induction, and rescue by IFN-pathway inhibition may therefore be needed to establish whether candidate compounds phenocopy genetic loss of EndoU function.

### Non-EndoU inhibitors

In contrast to classical active-site inhibitors, several compounds appear to target Nsp15 through mechanisms that do not rely on direct inhibition of EndoU activity. These studies suggest that perturbing Nsp15 structure, oligomerization, or functional state may be sufficient to inhibit coronavirus replication. Stevaert et al. identified 1,2,3-triazolo-fused betulonic acid derivatives with strong and selective anti-HCoV-229E activity, with compound 5h emerging as a lead molecule (110). Its activity was linked to Nsp15 by resistance mutations at K60R and T66I and reduced activity against an EndoU-deficient H250A mutant virus. Docking suggested that 5h may bind a pocket at the Nsp15 dimer interface adjacent to the active site of one protomer and the N-terminal domain of the neighboring protomer, supporting an interface-directed mode of targeting. This series was active against HCoV-229E but not SARS-CoV-2, consistent with incomplete conservation of the proposed pocket; follow-up SAR (structure–activity relationship) studies provided guidance for optimization but did not surpass the original lead (111).

Van Loy et al. reported EPB-113 as another Nsp15-targeting inhibitor with activity against HCoV-229E and SARS-CoV-2 (96). Resistance selection mapped to Nsp15, yielding G248V and H250Y substitutions in the EndoU catalytic core, while biophysical analyses showed that EPB-113 binds hexameric Nsp15, reduces its thermal stability, and unexpectedly enhances rather than inhibits EndoU cleavage. Although its antiviral activity is predominantly IFN-independent, recombinant viruses carrying EPB-113 resistance mutations show altered immune phenotypes and marked fitness costs, including impaired replication in human macrophages and reduced competitiveness relative to WT virus (96). Together, these studies indicate that Nsp15 can be targeted through mechanisms beyond active-site inhibition, including disruption of interprotomer interfaces, destabilization of the hexamer, and modulation of allosteric or structural states. Such approaches may be particularly valuable because Nsp15 appears to contribute to infection through both catalytic and non-catalytic functions, and because perturbing oligomeric organization may affect RNA binding, RTC-associated functions, and immune evasion simultaneously.

## UNRESOLVED QUESTIONS AND FUTURE PERSPECTIVES

**Fig 5:**
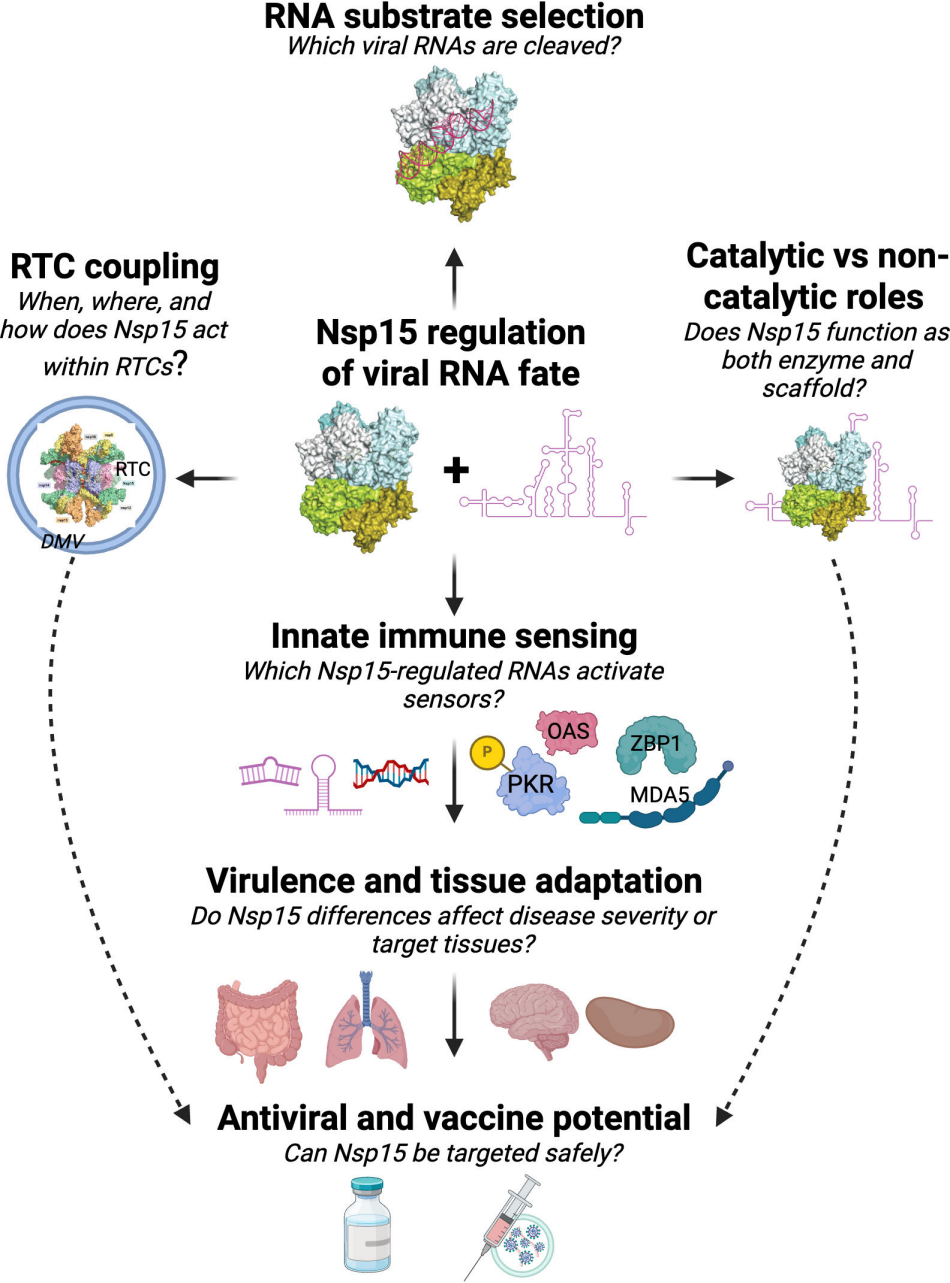
Unresolved questions and future directions in coronavirus Nsp15 biology. This schematic diagram highlights major gaps in understanding how Nsp15 regulates viral RNA fate and how this activity connects to RTC function, innate immune sensing, virulence, tissue tropism, and antiviral or vaccine development. Solid arrows indicate proposed biological relationships; dashed arrows indicate potential translational links. Figure created with BioRender.com.

Over the past decade, Nsp15 has evolved from a poorly characterized viral nuclease into a multifunctional regulator of viral RNA fate. Rather than acting solely as a degradative enzyme, accumulating evidence indicates that Nsp15 shapes the composition, structure, and immunogenic potential of viral RNA species. This expanded view provides a framework for understanding Nsp15 function but also highlights key unresolved questions (Fig. 5). These questions are interconnected: defining the physiological RNA substrates of Nsp15 will be essential for understanding its immune-evasion function, its potential role in RTC organization, and how it can be safely targeted therapeutically.

A central issue is the identity and selection of physiological RNA substrates. While biochemical and transcriptome-wide studies have identified preferential cleavage at U-A and C-A junctions, it remains unclear whether Nsp15 targets defined RNA classes or more broadly cleaves structurally accessible RNA within replication organelles. The relative contributions of PUN RNAs, structured UTR and TRS elements, and aberrant RNAs such as defective viral genomes remain to be determined. Closely related is how Nsp15 distinguishes RNA species that must be preserved for replication from those targeted for cleavage. Whether selectivity is governed by RNA structure, local accessibility, spatial constraints within DMVs, or coordination with RNA synthesis remains unclear. Addressing this will require integration of RTC structural studies with genome-wide mapping of RNA cleavage in physiologically relevant systems.

Another challenge is understanding how catalytic and non-catalytic functions are integrated. Although EndoU activity is dispensable for RNA synthesis *per se* in several systems, structural integrity, oligomerization, and RTC interactions can be required for replication. Thus, Nsp15 may function both as an enzyme and as a structural or organizational component of the replication machinery. The relationship between RNA processing and innate immune modulation also remains incompletely defined. Nsp15 limits activation of MDA5, PKR, OAS/RNase L, and ZBP-1, but the RNA species that trigger each pathway and how they are generated remain unclear. In addition, Nsp15 may influence broader RNA-driven host responses, including translation, stress signaling, RNA stability, programmed cell death, and STING activation.

An additional question is whether differences among coronavirus Nsp15 proteins contribute to differences in virulence. Because EndoU activity suppresses innate immune activation, variation in catalytic activity, substrate specificity, interactions with the RTC, or regulatory mechanisms could influence pathogenic potential. However, current evidence does not support a simple model in which Nsp15 proteins from highly pathogenic CoVs are intrinsically stronger immune antagonists than those from less pathogenic CoVs. Rather, the impact of Nsp15 on virulence appears to depend on viral species and strain, target organs and cell types, and coordination with other immune antagonists, as illustrated by MERS-CoV. Direct comparative studies will be needed to determine whether natural variation in Nsp15 activity or substrate targeting influences virulence, tissue tropism, and host adaptation.

From a translational perspective, Nsp15 presents both opportunities and challenges. Catalytic inhibitors often show measurable biochemical activity but limited cellular antiviral effects, likely reflecting insufficient potency, specificity, target engagement, and the use of immune-deficient systems. Non-catalytic inhibitors that perturb Nsp15 structure or oligomeric state provide proof-of-concept for alternative targeting strategies. A key question is whether Nsp15 can be modulated to enhance antiviral immunity without triggering excessive inflammation or immunopathology. Similarly, immune-dependent attenuation of EndoU-deficient viruses highlights the potential of Nsp15 for rational live-attenuated vaccine design (59, 97, 112), but variable attenuation across viruses and hosts suggests that Nsp15-based attenuation will likely need to be most effective as part of a multi-layered strategy incorporating additional safeguards to ensure genetic stability and safety.

In summary, Nsp15 is best understood not simply as an endoribonuclease but as a dynamic regulator of viral RNA fate that integrates RNA processing, replication, and immune evasion. Addressing the questions outlined above will require coordinated structural, biochemical, and systems-level approaches. A more complete understanding of how Nsp15 shapes the viral RNA landscape will refine current models of coronavirus biology and inform antiviral and vaccine strategies.
